# Waiting for family reunification and the risk of mental disorders among refugee fathers: a 24-year longitudinal cohort study from Denmark

**DOI:** 10.1007/s00127-021-02170-1

**Published:** 2021-09-05

**Authors:** Camilla Hvidtfeldt, Jørgen Holm Petersen, Marie Norredam

**Affiliations:** 1 Danish Research Centre for Migration, Ethnicity and Health; Section of Health Services Research, Department of Public Health, University of Copenhagen, Copenhagen, Denmark; 2 The ROCKWOOL Foundation Research Unit, Copenhagen, Denmark; 3 Section of Biostatistics, Department of Public Health, University of Copenhagen, Copenhagen, Denmark

**Keywords:** Refugees, Family separation, Family reunification, Mental disorders, Cohort, Longitudinal

## Abstract

**Purpose:**

To examine whether family separation caused by prolonged waiting for family reunification is associated with the risk of mental disorders among refugee fathers.

**Method:**

Based on full-population Danish registry data covering 1995–2015, we mapped arrival patterns among nuclear refugee family members resettled in Denmark (*n* = 76,776) and established a cohort of refugee fathers (*n* = 6176) who all arrived alone and later obtained family reunification with their wife and children. The fathers were followed for up to 24 years, from the day their residence permit was issued until their first psychiatric diagnosis, emigration, death, or study end, whichever came first. Using Cox proportional hazard regression, we estimated hazard ratios (HRs) of being diagnosed with a mental disorder (i) for the period while the fathers were still separated from their family and (ii) across varying lengths of family separation.

**Results:**

The HR of any mental disorder was 2.10 (95% confidence interval (CI): 1.57–2.81) for fathers still separated from their family compared with those who had obtained family reunification. The HR increased with longer family separation. Compared with fathers separated for < 9 months, the HR of any mental disorder was 1.43 (95% CI 1.08–1.89) for 9–11 months’ separation, increasing to 2.02 (95% CI 1.52–2.68) for 18–23 months’ separation. Results were driven by post-traumatic stress disorder.

**Conclusion:**

Fathers waiting for their wives and children face an increased risk of mental disorders. Countries receiving refugees should be aware that delaying family reunification can lead to adverse mental health effects.

**Supplementary Information:**

The online version contains supplementary material available at 10.1007/s00127-021-02170-1.

## Introduction

Family separation is a risk factor of mental health problems—in general, and more specifically for migrants, refugees and their families [1–6]. Nevertheless, many families experiencing war and disaster feel compelled to separate and let one or a few family members travel ahead of the others to seek asylum and, if they are recognised as refugees, to apply for family reunification. In Europe, most asylum seekers are men, while most family reunified to refugees are women and children [7, 8]. This indicates that the ‘first arrivals’ are often fathers who begin their life in the receiving country waiting for their families. The fathers do not know how long it will take to process their applications, first for asylum and then, for family reunification, neither the outcome of the applications. Hence, they wait with ‘double uncertainty’. Such waiting with double uncertainty can increase the risk of mental disorders [9, 10].

During the last decade, many Western countries have implemented immigration policies and practices that extend the family reunification process, thereby prolonging family separation periods. One example of such policy is the suspension of the right to seek family reunification for refugees with subsidiary or temporary protection status implemented in, for example, Germany, Denmark, and Sweden [11]. Another example is the compulsory collection of DNA tests used in the United States and many other Western countries to ascertain biological relationships between relatives [12–14]. Such tightening of laws and administrative procedures increases the importance of identifying potential adverse effects of prolonged periods of waiting for family reunification on refugees’ mental health.

Research has indicated that family separation is associated with increased risk of depression, anxiety, suicide, and post-traumatic stress disorder [2, 3, 15–22]. Refugees waiting for their families have been shown to feel powerless, distressed, and guilty for being safe while their family remain in potential danger [1, 2, 23, 24]. Put together, the findings suggest that family separation may add cumulatively to the stressors and trauma experienced by most refugees, thereby aggravating their mental health problems [2, 25, 26]. However, high-quality research on the relationship between the time spent waiting for family reunification and mental health is sparse. Among the few studies that focus on family separation, most are based on smaller non-representative, cross-sectional samples, and do not account for the non-randomness of family separation [1, 20, 21, 23, 24]. We have not been able to identify any longitudinal, cohort study that examined the association between the length of the family separation period and the risk of mental disorders among adult refugees.

To fill these gaps, in this study, we aimed to examine the association between family separation and the risk of mental disorders among a large cohort of refugee fathers. To make the included refugees highly comparable and account for selection issues, we identified all refugee nuclear families resettled in Denmark since 1995 and mapped the arrival patterns among the family members. Thereby, we were able to establish a cohort of refugee fathers who all arrived without their family and hence were first separated from their family and then later reunified. We addressed three questions: First, do refugee fathers still waiting for family reunification have a higher risk of mental disorders compared with those who have been reunified with their family? Second, are longer periods of family separation associated with a higher risk of mental disorders? Finally, is prolonged family separation associated with an increased risk of mental disorders even after family reunification has been obtained?

## Methods

### Study design and participants

The study population included refugee fathers from nuclear families with at least one child younger than 18 years (*n* = 6176). The fathers should (a) be ‘first arrivals’, i.e., the first within their family to settle in Denmark, (b) have arrived between 1 January 1995 and 31 December 2015, (c) hold a convention or protection refugee status, and (d) have obtained family reunification with their wife and children after having received their residence permit. A short description of the Danish context is provided in the Supplementary Material.

**Fig. 1:**
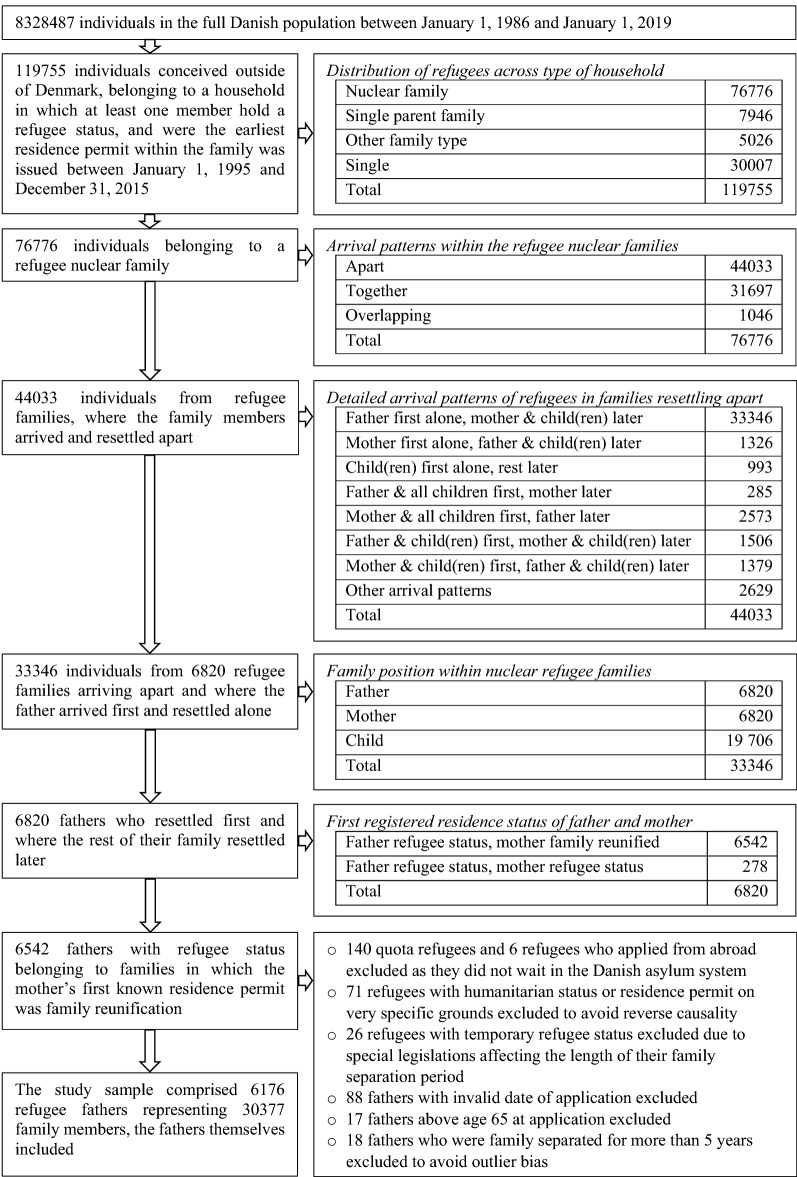
Flowchart, selection of study sample and distributions across types of households and arrival patterns

To identify the family units, we searched Danish full-population, administrative data covering 1 January 1986 to 31 December 2019 (*n* = 8,328,487). The search for family members covered a longer period than that where the fathers were included to ensure that the fathers were the first within their families to arrive and had time to obtain family reunification. All included fathers were apart from their family at study start and were reunified with their family before 31 December 2019. We used the unique Danish personal identification numbers to link spouses and to link parents to children [27]. Hence, families were defined by spouse and parent–child relationships. We identified all families with at least one member having refugee status and determined (i) the type of family (nuclear family, single-parent family, and other), (ii) the arrival pattern within the family (apart, together, and overlapping), and (iii) the individuals’ position within the families (father, mother, child). Accordingly, we were able to select refugee fathers fulfilling the aforementioned inclusion criteria. The flowchart in Fig. 1 illustrates the selection of the study sample, including distributions across types of households and arrival patterns.

### Measures

#### Mental disorders

The outcome variable was first-time primary psychiatric hospital diagnosis obtained through the Danish National Patient Register [28]. Diagnoses were based on the International Classification of Diseases, tenth revision (ICD-10). Emergency room diagnoses were excluded. We investigated the risk of ‘any’ mental disorder, including F2–F4, and conducted separate analyses for the associations with family separation for the three most frequent disorders: psychotic disorders (F20–29), affective disorders (F30–F39), and neurotic and stress-related disorders (F40–F48). The first-time diagnosis date was registered separately for each diagnosis. Hence, the survival time varies across diagnosis. Mental health problems during the asylum phase, diagnoses by private specialists in psychiatry, and treatment of mild to moderate mental illness by general practitioners were not registered in the data [29]. This implies that only the most severe ends of the spectra were captured.

#### Exposure variables

**Fig. 2:**
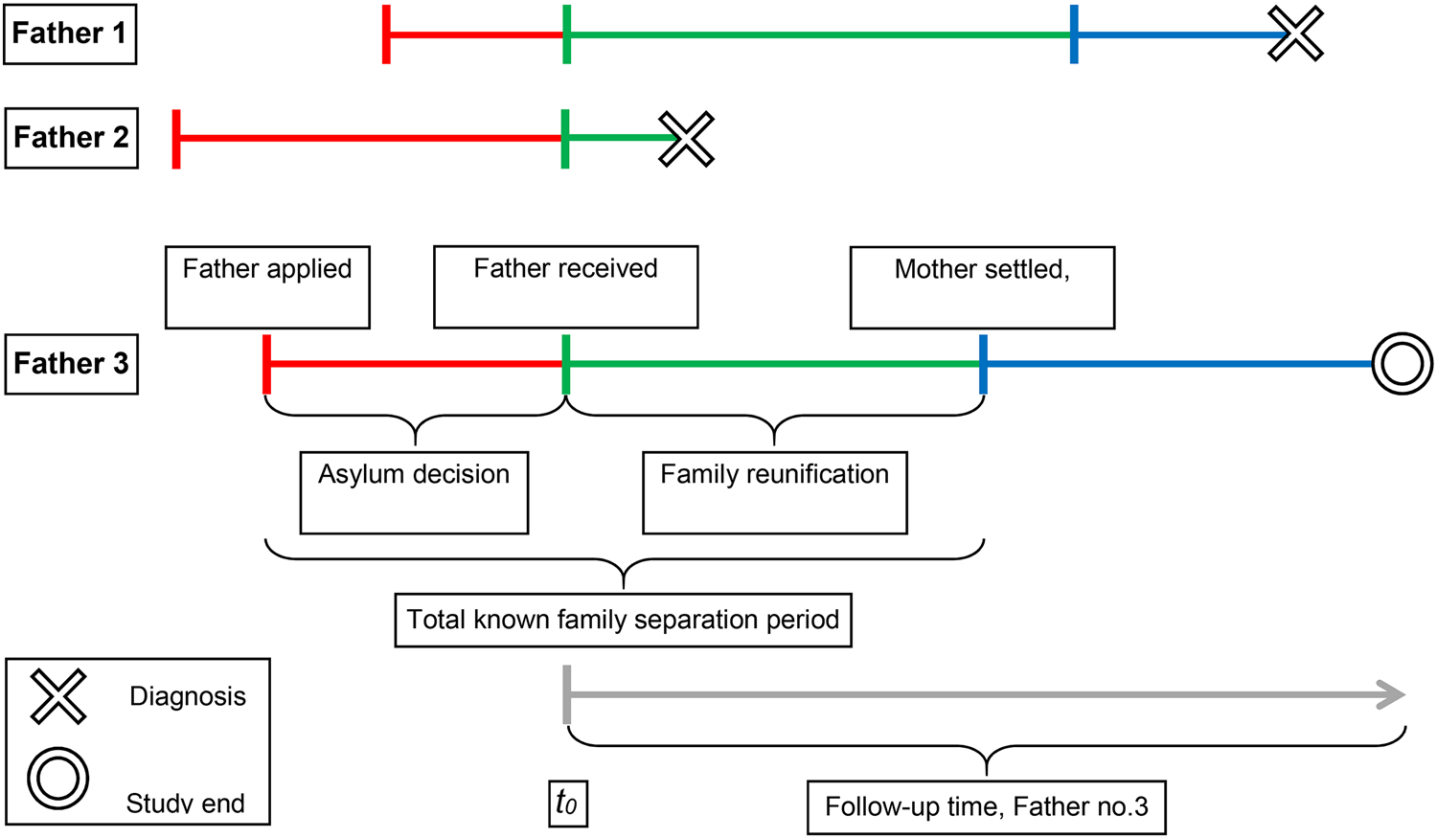
Composition of the known family separation period for three refugee fathers. The total known family separation period is the sum of two intervals: the asylum decision waiting time (red line) and the family reunification waiting time (green line). The blue line marks the time after family reunification. Follow-up starts when the father receives a residence permit (time *t*_*0*_). Father no. 1 spent shorter time waiting for an asylum decision than Father no. 2 and 3, he was diagnosed with a psychiatric disorder after being reunified with his family. Father no. 2 was diagnosed before his family arrived, whereas Father no. 3 was followed until the study end without being diagnosed. The grey line illustrates the follow-up period of Father no. 3

The exposure variable, the length of the family separation period, was examined in three different ways. First, by including a measure for the total known family separation computed as the difference between the father’s date of asylum application and the date his wife was registered as settled in a Danish municipality (see Fig. 2). Second, by dividing the total known family separation period into asylum decision waiting time and family reunification waiting time and including measures for both periods. This allowed us to evaluate if the associations with mental disorders differed for the two types of waiting. Third, by splitting the first 30 months of the follow-up period into the intervals 0–5, 6–8, 9–11, 12–17, 18–23, and 24–30 months and in each period comparing the risk of mental disorder among fathers who were still separated with those who had obtained family reunification. Thereby we could examine if the association between family separation and mental disorders changed markedly during the first 30 months after resettlement.

To take into account that the fathers’ status changed from ‘separated’ to ‘reunified’ when they obtained family reunification, we constructed several time-dependent variables. First, we created a dummy variable that was coded 1 while the family remained separated and 0 after family reunification. The dummy variable assesses the ‘temporary risk’ of being diagnosed with a mental disorder while still waiting for family reunification by comparing the risk of separated fathers with the risk of those already reunified. This dummy variable was included in all analyses.

Second, to investigate whether longer periods of family separation were associated with a higher risk of mental disorders, we constructed two categorical, time-dependent variables. The one categorical variable, included in the analysis of the total known family separation period, changed values after 9, 12, 18, and 24 months of family separation. To consider that the family reunification waiting time was shorter than the total known family separation, the other categorical variable, included in the analyses where the total known family separation time was divided into asylum waiting time and family reunification waiting time, changed values after 6, 9, and 12 months of waiting. Finally, the asylum decision waiting time was categorised into 0–2, 3–5, 6–11, and 12–60 months of waiting and included as a time-independent variable in analyses of the divided family separation period. Because all these categorical variables regard periods both before and periods after family reunification, and to discern them from the ‘temporary risk’ while still separated, we term the associations they measure as ‘longer-term risk’ of mental disorders.

#### Covariates

Age at application (18–29 years, 30–39 years, 40–65 years), cohort of application (1991–2001, 2002–2015), region of origin (Middle East and North Africa, Sub-Saharan Africa, and others), and a dummy for settlement in the Danish province ‘North Jutland’ were used for stratification. Provinces of first settlement in Denmark (Copenhagen city (capital), Copenhagen suburb, North Zealand, Bornholm, East Zealand, West & South Zealand, Funen, South Jutland, East Jutland, and West Jutland) were included as confounders. Number of children in the family upon resettlement and type of refugee status (convention or protection) were insignificant and omitted. The quality of information on migrants’ educational level is low compared with other Danish administrative data and educational level is imputed or missing for approximately a third of the sample. Imputed values are based on both pre- and post-migration factors [30, 31]. Hence, to avoid introducing endogeneity in the analyses, education was only included in a sensitivity analysis.

Information on dates of application and residence permits was delivered by the Immigration Service, while all other information was made available by Statistics Denmark.

### Statistical analysis

We used Cox proportional hazard models to estimate hazard ratios (HRs) with 95% confidence intervals (CIs) for the risk of psychiatric diagnosis. All included fathers were apart from their family at study start and reunified before 1 January 2019. Follow-up started at the fathers’ first registered date of residence permit as we have no information on their health status prior to this date. The entry period was between 1 January 1995 and 31 December 2015. Follow-up ended on the date of first psychiatric diagnosis, first emigration from Denmark, death, or at study end (27 June 2019), whichever came first. All events were registered on dates.

The statistical model applied assumes independent censoring and proportional hazard (PH) for the exposures and covariates. The independent censoring assumption were not considered to be violated because only a minority of the participants emigrated or died during follow-up. The PH assumption was tested based on Schoenfeld residuals. First, we investigated the unadjusted correlations between time and Schoenfeld residuals for each variable in the study. Second, we tested for nonzero slopes of the scaled Schoenfeld residuals from the main models, using both a global test and tests for the individual covariates (rank transformed) [32]. The PH assumption was violated for age at application, cohort of application, region of origin, and settlement in North Jutland. Consequently, analyses were stratified on these variables. Beyond stratification, main analyses were adjusted for Danish provinces, origin in Middle East and ‘other origin’. Supplementary Table 3 shows parameters from an unstratified model.

### Sensitivity analyses

We also tested for interactions. There was no interaction between time waiting for, respectively, asylum and family reunification and thus the two types of waiting did not modify each other. Additionally, we tested for time-variance by splitting the follow-up after family reunification was obtained into 0–5, 5–10, and 10–24 years (Supplementary Material Table 4) and by including interactions between survival time and the two types of waiting (Supplementary Material Table 5). The association between the risk of mental disorders and waiting for family separation became statistically insignificant in follow-up periods longer than 5 years but the interaction parameters between time and the waiting time variables were all statistically insignificant. Moreover, we performed analyses with biological age as the underlying time scale instead of time since residence permit issuance (Supplementary Material Table 6). This did not alter the main conclusions except for the risk of affective disorder which became statistically significant during the separation period. PH assumptions were not fulfilled for two out of four models with age as time scale, therefore models with time since residence permit issuance as underlying time scale are preferred. Finally, we performed a sensitivity analysis including a variable for no or some education and a dummy for imputation (see Supplementary Material Table 7). This analysis did not alter the conclusions.

All analyses were conducted using Stata 15.1.

### Data availability

This study was based on semi-anonymised data from Statistics Denmark and the Danish Immigration Service. To gain access to the data, researchers need to be affiliated to a Danish authorised research environment. Authorisation is undertaken by Statistics Denmark (see https://www.dst.dk/en/TilSalg/Forskningsservice). Further, the Danish Immigration Service must agree on granting access to data on refugees’ data of application and residence status.

### Ethical approval

The study was approved by the Danish Data Protection Agency and by Statistics Denmark, which made the data available with encrypted personal identification numbers. According to Danish legislation, no further consent was required.

## Results

The inclusion criteria were met by 6176 refugee fathers. Of the fathers, 1219 (21.8%) received a psychiatric diagnosis during follow-up (see Table 1), 112 were diagnosed with a mental disorder, 227 with an affective disorder, and 1073 with a neurotic or stress-related disorder, of whom 943 (88%) were diagnosed with post-traumatic stress disorder (PTSD); 179 had more than one diagnosis. Most of the fathers (77.2%) experienced more than 1 year of total family separation; 15.3% waited longer than 1 year for the asylum decision, while 40.4% waited longer than 1 year to be reunified with their family after receiving their own residence permit (Supplementary Material Fig. 1 shows the distributions of the waiting periods). The incidence rate (IR) per 1000 person-years for any mental disorder was 39.3 while family separated and 19.6 after family reunification. Mean follow-up was 9.1 years. During the follow-up period, 83 (1.5%) fathers died and 416 (7.4%) emigrated.

**Table 1:** Cohort characteristics^a^ at start and end of follow-up, refugee fathers resettled in Denmark 1995–2015

	Population, study start		Population, study end		Person- years	Any mental disorder		Deaths		Emigrations	
	*n*	%	*n*^b^	%^b^	PY	*n*	IR	*n*^c^	IR	*n*^c^	IR
Family separation status											
Separated	6176	100	5921	95.9	6236	245	39.3	< 3	< 0.5	9–11	1.4–1.8
Reunified	0	0	4458	75.3	49,708	974	19.6	81–83	1.6–1.7	405–407	8.1–8.2
Total known family separation period											
0–8 months	418	6.8	339	81.1	2816	49	17.4	4	1.4	26	9.2
9–11 months	966	15.6	762	78.9	6151	151	24.5	9	1.5	44	7.2
12–17 months	2058	33.3	1521	73.9	17,354	382	22.0	20	1.2	135	7.8
18–23 months	1184	19.2	765	64.6	12,775	299	23.4	20	1.6	100	7.8
24–90 months	1550	25.1	1071	69.1	16,848	338	20.1	30	1.8	111	6.6
Family reunification waiting time											
0–5 months	554	9.0	378	68.2	7369	109	14.8	14	1.9	53	7.2
6–11 months	3128	50.6	2266	72.4	27,502	646	23.5	36	1.3	180	6.5
12–60 months	2494	40.4	1814	72.7	21,074	464	22.0	33	1.6	183	8.7
Asylum decision waiting time											
0–2 months	1959	31.7	1621	82.7	8993	251	27.9	6	0.7	81	9.0
3–5 months	1430	23.2	1067	74.6	10,984	238	21.7	18	1.6	107	9.7
6–11 months	1841	29.8	1198	65.1	23,447	454	19.4	38	1.6	151	6.4
12–60 months	946	15.3	572	60.5	12,520	276	22.0	21	1.7	77	6.2
Age at application											
18–29 years	1168	18.9	856	73.3	11,095	189	17.0	5	0.5	118	10.6
30–39 years	3065	49.6	2231	72.8	26,332	605	23.0	21	0.8	208	7.9
40–65 years	1943	31.5	1371	70.6	18,517	425	23.0	57	3.1	90	4.9
Period of application											
1991–2001	2653	43.0	1571	59.2	40,131	721	18.0	73	1.8	288	7.2
2002–2015	3523	57.0	2887	81.9	15,813	498	31.5	10	0.6	128	8.1
Geographical origin											
Middle East & North Africa	5214	84.4	3770	72.3	46,205	1118	24.2	69	1.5	257	5.6
Sub-Saharan Africa	769	12.5	557	72.4	7198	52	7.2	11	1.5	149	20.7
Other	193	3.1	131	67.9	2541	49	19.3	3	1.2	10	3.9
Danish province of resettlement											
Copenhagen (capital)	196	3.2	131	66.8	2721	29	10.7	10	3.7	26	9.6
Copenhagen suburb	163	2.6	115	70.6	1737	29	16.7	5	2.9	14	8.1
North Zealand	573	9.3	412	71.9	5028	123	24.5	7	1.4	31	6.2
East Zealand	314	5.1	223	71.0	2832	68	24.0	3	1.1	20	7.1
West & South Zealand	889	14.4	606	68.2	7858	206	26.2	7	0.9	70	8.9
Funen	650	10.5	469	72.2	5840	122	20.9	7	1.2	52	8.9
South Jutland	924	15.0	713	77.2	7092	160	22.6	8	1.1	43	6.1
East Jutland	937	15.2	653	69.7	7993	201	25.1	17	2.1	66	8.3
West Jutland	705	11.4	514	72.9	6792	145	21.3	8	1.2	38	5.6
North Jutland	825	13.4	622	75.4	8050	136	16.9	11	1.4	56	7.0
Total	6176	100.0	4458	72.2	55,944	1219	21.8	83	1.5	416	7.4

^a^Distribution of population, person-years (PY), cases (*n*), and unadjusted incidence rates (IR) per 1000 person-years for any mental disorder, death, and emigration

^b^Out of population at study start. For “Separated” the number and percentage regard the end of the family separation period; for “Reunified” the percentage is out of population at study start

^c^To secure anonymity of data, Statistics Denmark demands *n* < 3 to be masked and as the ns in columns 3, 6, 8, and 10 sums to the population at study start (column 1), small ns result in two masked cells

Syrians constituted 91% of the fathers who waited 0–2 month for an asylum decision and 78% of the 2002–2015 cohort. Syrians also had one of the highest IRs of any mental disorder (see Supplementary Table 1). These patterns explain the high IR (27.9) among those with shortest time waiting for an asylum decision as well as the high IR in the 2002–2015 cohort. In general, the IRs increased with longer waiting times for both Syrians and non-Syrians (see Supplementary Table 2).

Higher age was associated with higher IRs of mental health problems. This is to some degree explained by older fathers tending to have more children. Older fathers may also on average have older children with whom they cannot obtain family reunification, which may further add to their psychological burden. Finally, Table 1 demonstrates lower IRs in the Copenhagen city and suburb. This is partially a result of the Danish dispersal policies which caused the capital area to receive fewer refugees during 2002–2015 when the IRs were higher [33].

Table 2 presents the hazard ratios with 95% confidence intervals (CI) for different mental disorders. Model 1–4 presents estimates for the total known family separation period. Fathers who were still waiting from their family had significantly higher risk of being diagnosed with any mental disorder compared with fathers who had been reunified (hazard ratio 2.10, 95% CI 1.57–2.81). The temporary risk was only increased for neurotic and stress-related disorders (hazard ratio 2.09, 95% CI 1.53–2.85) not for psychotic or affective disorders. The longer-term risk of any mental disorder increased with length of family separation (Model 1). Compared with those separated for 0–8 months, the hazard ratio was 1.43 (95% CI 1.08–1.89) for fathers separated for 9–11 months and 1.86 (95% CI 1.39–2.48) for fathers separated for 24–90 months. The patterns for longer-term risk of neurotic and stress-related disorders resembled those of any mental disorder (Model 4), whereas the longer-term risk of psychotic and affective disorders did not increase with length of total known family separation (Models 2 and 3).

**Table 2:** Refugee fathers' risk of mental disorders^a^ across varying lengths and types of family separation^b^

	Any mental disorder		Psychotic disorders		Affective disorders		Neurotic & stress-related disorders	
Model 1–4	HRs	95% CI	HRs	95% CI	HRs	95% CI	HRs	95% CI
Family separation								
Reunified (ref.)	1.00		1.00		1.00		1.00	
Separated	2.10***	1.57–2.81	2.44	0.91–6.56	1.43	0.67–3.06	2.09***	1.53–2.85
Total known family separation period								
0–8 months (ref.)	1.00		1.00		1.00		1.00	
9–11 months	1.43*	1.08–1.89	1.15	0.35–3.80	1.08	0.54–2.17	1.56**	1.16–2.10
12–17 months	1.56**	1.20–2.04	1.28	0.43–3.75	1.73	0.92–3.27	1.62***	1.22–2.17
18–23 months	2.02***	1.52–2.68	1.69	0.57–5.07	1.53	0.78–3.01	2.12***	1.57–2.88
24–90 months	1.86***	1.39–2.48	1.50	0.50–4.54	1.34	0.67–2.67	1.99***	1.46–2.72
Model 5–8								
Family separation								
Reunified (ref.)	1.00		1.00		1.00		1.00	
Separated	2.25***	1.67–3.03	2.74	1.00–7.52	1.64	0.75–3.56	2.22***	1.61–3.04
Asylum decision waiting time								
0–2 months (ref.)	1.00		1.00		1.00		1.00	
3–5 months	1.28*	1.06–1.54	1.59	0.66–3.83	1.33	0.82–2.15	1.29*	1.06–1.56
6–11 months	1.66***	1.35–2.05	2.26	0.90–5.66	2.07**	1.23–3.49	1.58***	1.27–1.97
12–60 months	1.85***	1.47–2.33	1.87	0.71–4.92	2.55***	1.46–4.46	1.70***	1.33–2.17
Family reunification waiting time								
0–5 months (ref.)	1.00		1.00		1.00		1.00	
6–8 months	1.39**	1.10–1.76	1.25	0.61–2.53	1.47	0.91–2.39	1.44**	1.11–1.85
9–11 months	1.48**	1.17–1.88	1.36	0.66–2.79	1.21	0.73–2.01	1.61***	1.24–2.09
12–60 months	1.38**	1.09–1.74	1.17	0.58–2.38	0.93	0.56–1.55	1.51**	1.17–1.94
No. of diagnosed fathers	1219		112		227		1073	

^a^Diagnoses based on first-time hospital contact for 6176 refugee fathers resettled in Denmark 1995–2015

^b^Models 1–4: Varying lengths of total known family separation. Models 5–8: Family separation divided into asylum decision and family reunification waiting time. Results are displayed as hazard ratios (HRs) with 95% confidence intervals (CIs). Survival time starts when the fathers receive their residence permit. Analyses are adjusted for Danish provinces and origin in Middle East or 'other regions', and stratified on age at application (18–29, 30–39, and 40–65 years), period of application (before or after 2001), Sub-Saharan origin, and settlement in province North Jutland

*P < 0.05, **P < 0.01, ***P < 0.001

Models 5–8 in Table 2 show the estimates for the risk of mental disorders for the total known family separation divided into asylum decision and family reunification wait time. The estimates for the temporary risks of the different mental disorders were similar to those of Model 1–4. Concerning the longer-term risk of any mental disorder (Model 5), the analyses demonstrate that protracted waiting was linked to a higher risk, regardless of whether the waiting was for asylum or for family reunification. As to waiting for family reunification, the hazard ratio was around 1.4 disregarding of the length of the waiting; hence, there was no sign of exacerbation for longer periods of waiting for family reunification. By contrast, the hazard ratios increased numerically with longer periods waiting for asylum, from 1.28 (95% CI 1.06–1.54) for 3–5 months of waiting to 1.85 (95% CI 1.47–2.33) for 12–60 months of waiting. The analyses with the family separation period divided into time waiting for, respectively, an asylum decision and family reunification revealed that the longer-term risk of affective disorders increased with longer asylum decision wait time but not with prolonged time waiting for family reunification (Model 7).

Analyses of the longer-term risk of any mental disorder assessed for the periods 0–24, 0–5, 5–10, and 10–24 years after the fathers were reunified with their families revealed a numerically higher risk in all follow-up periods; however, the estimates were not significant for periods longer than 5 years after family reunification (Supplementary Material Table 4).

**Fig. 3:**
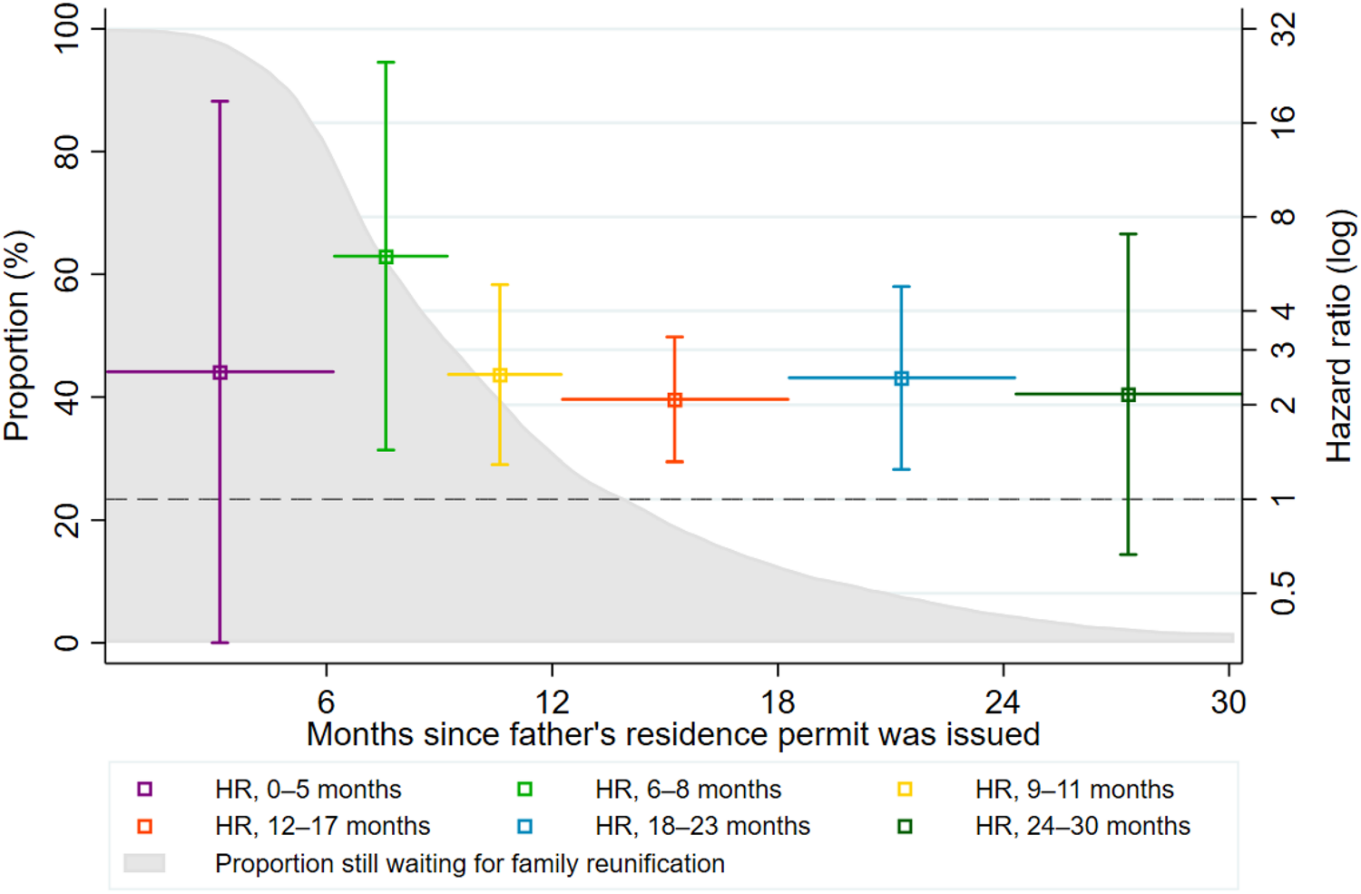
The proportion still waiting for family reunification (grey) of those without a diagnosis at time t, and the HRs for the risk of mental disorder (with 95% confidence intervals) for fathers still separated compared with those who were already reunified across different intervals of the first 30 months after residence permit issuance

Lastly, Fig. 3 shows two elements: first, the proportion still awaiting family reunification (marked with grey) out of those without a diagnosis at different times of the first 30 months of the follow-up; second, the estimates (with 95% CIs) of any mental disorder for fathers still separated compared with those who have been reunified in particular periods. For example, among fathers who received their residence permit 12–17 months earlier, and who had not been diagnosed until the time of observation, the HR of any mental disorder was 2.1 [1.3–3.3]. The figure demonstrates that almost all fathers had been reunified with their families 30 months after their residence permits were issued, and that the association between family separation and the risk of being diagnosed with a mental disorder was relatively constant while waiting for family reunification.

## Discussion

The present study examined the association between family separation and the risk of mental disorders. Using full-population Danish registry data we established a cohort of 6176 refugee fathers who all arrived alone and later obtained family reunification. Our findings demonstrated that the fathers’ risk of mental disorders was more than twice as high when separated from their families compared with those who had already obtained family reunification. We also showed that the risk of mental disorders increased with longer periods of waiting for family reunification, and that the risk was increased even after family reunification was obtained. The results suggested that family separation was mainly associated with an increased risk of PTSD.

To the best of our knowledge, this is the first large-scale, cohort study to demonstrate that family separation is associated with an increased risk of mental disorders among refugee fathers. Our study adds to previous findings in several ways. First, in contrast with earlier studies, we established a cohort of refugees fathers who were followed for a long period after their resettlement, where the fathers were comparable in the sense that they were all part of a nuclear family before application for asylum, all arrived alone, and all were later reunified with their family [1, 15, 20, 23, 34]. Second, we provided a reliable estimate of the magnitude of the risk of being diagnosed with a mental disorder while the fathers were still separated from their families. Third, we showed that prolonged family separation represented a risk factor for developing mental disorders, both during the family separation period and after family reunification. For instance, the longer-term risk of being diagnosed with a mental disorder was more than 80% increased among fathers who experienced a total known family separation period for more than 17 months compared with those who were separated for less than 9 months. Finally, we made a conceptually clear division of the total known family separation period into asylum decision and family reunification wait time and demonstrated that both types of waiting contributed to the increased risk of mental disorders.

Our results can be explained by several factors. First, when waiting for an asylum decision or for family reunification the refugee fathers experience a ‘double uncertainty’ because they neither know whether their application will be approved nor when the application process will be over. Double uncertainty represents a stressor that can impede the ability to correct negative beliefs and hinder recreation of a coherent autobiography that integrates potentially traumatic events [25]. Moreover, waiting with double uncertainty may induce a more atomistic thinking where the fathers’ time perception splits in ‘before’ and ‘after’ the application decisions and the intermediate periods ‘collapse’ [35]. This can further prevent refugees from contextualising traumatic experiences, increase the risk of re-traumatisation, and hinder recovery. Second, it has been documented that refugees fear for the safety of their family when they experience extended family separation [23]. Fear is in itself an additional stressor that can have adverse mental health effects [20]. A third and related factor concerns the family position of the individuals in the study population: they are all fathers and husbands. In many cultures, fathers are traditionally supposed to be the head of the family and to protect family members. Being barred from undertaking this function and not being able to affect the length of the waiting period may add to the feelings of powerlessness described in the literature [20, 36].

Previous studies have focused on protracted waiting for asylum [9, 10]. But often the average family reunification waiting time exceeds the time waiting for asylum. In our study, the association between length of family separation and risk of mental disorders was of similar magnitude to the one between asylum decision wait time and risk of mental disorders. If the period waiting for family reunification exceeds the period waiting for an asylum decision, it makes prolonged family separation a larger problem than long asylum decision processes. Therefore, the results of this study underline the necessity of considering both types of waiting when evaluating the potential effects of immigration policies.

The study results were mainly driven by an increased risk of developing PTSD. This is in accordance with previous research that found waiting for an asylum decision to increase the risk of PTSD [9]. Regarding affective disorders such as depression, these were predicted by prolonged periods of asylum decision wait time but not by the length of the family reunification wait time. This is surprising because affective disorders share many features with neurotic and stress-related disorders and are often comorbid with PTSD [37, 38]. The result may be explained by lack of access to diagnoses from the general practitioners who treat mild to moderate depression with antidepressants.

### Strengths and limitations

The key strengths of this study relate to the individual level, cohort registry data. First, the data included links between family members, which allowed for construction of nuclear family units and mapping of the within-family arrival pattern. This enabled identification of highly comparable refugee fathers, and hence, greatly reduced potential selection bias. Second, information on the exact dates of key events, from birth to death, enabled precise computation the survival times, the total known family separation period, and the division of the known family separation period into waiting for asylum and family reunification. Third, we could control for various confounders by linking different registries. Finally, instead of self-reported data, we could use psychiatrists’ more valid primary diagnoses on mental disorders.

Our study also has several limitations. First, we did not have access to data from general practitioners and our analyses therefore only cover the most severe cases. Depression is often treated with anti-depressants by general practitioners. Hence, this limitation may explain the absence of an association between length of family reunification waiting time and affective disorders. Second, despite the high quality of the data, we were not able to establish a causal relationship because we could not establish variation in data that was unrelated to our exposure variables (such as legal amendments). Therefore, the presented links between family separation and increased risk of mental disorders are all to be interpreted as associations. Third, we could not identify fathers whose application for family reunification was rejected. Although this limitation does not affect our results, it is highly likely that the risk of mental disorders is higher for fathers who are denied family reunification than for the examined group of fathers. Additionally, unobserved mental illness on arrival, lack of resilience, and poor ability to adapt to the Danish society may all introduce reverse causality. These phenomena probably prolong the waiting periods, especially the time waiting for family reunification, because the fathers themselves must complete the complicated application for family reunification. Vulnerable fathers will find this task particularly difficult. In such cases, lengthy periods of waiting for family reunification could reflect poor mental health upon arrival, not vice versa, and induce upward bias. Moreover, we could not identify extended family members and therefore used a Western concept of family, focusing on nuclear family members. The presence of an unobserved extended family can induce upward bias if this both protects against mental illness and shortens the family separation period by providing advice about how to apply for family reunification. Another limitation is that we did not observe the date of application for family reunification. Some fathers may decide to postpone the application for family reunification to prepare for the family’s arrival if their wife and children are not immediately endangered. This could create downwards bias because the length of these fathers’ family separation would be prolonged, while they would be less anxious than those whose family remained in troubled areas. Lastly, family members’ unregistered settlement may also induce downwards bias as the length of the family separation period would be overestimated while the family was relatively safe and, accordingly, the risk of mental disorders lower. However, the latter phenomenon is unlikely to jeopardize the results because, if discovered, illegal residence in Denmark leads to all family members losing the possibility of obtaining permanent legal residence.

## Conclusion

The current study represents the most compelling empirical evidence to date supporting the notion that family separation can harm refugees’ mental health. Refugee fathers awaiting family reunification face an increased risk of being diagnosed with a mental disorder, not only while waiting for their family but also after the family has arrived. Clinicians and practitioners meeting and treating refugee fathers should be aware of the stress of family separation. They could consider establishing networks to support the fathers during the separation period. Even after reunification is obtained it is important to pay attention to family conflicts and potential limitations in the fathers' ability to support their children. Optimally, the reconciling and healing process should include all family members.

Poor mental health can spill over into worsened labour market attachment, making family poverty more likely. On a long-term perspective, parental mental health problems are found to be associated with increased risk of mental health problems and lower school performance among refugee children. Such potential costs should be considered when implementing policies prolonging family separation periods. Future research should seek to obtain information about refugees’ health status on arrival to explore heterogeneous effects of waiting. Furthermore, there is an urgent need to explore the mental health consequences for refugee fathers who are denied family reunification.

## Supplementary Information

Below is the link to the electronic supplementary material.

Supplementary file1 (DOCX 2950 KB)
