# Supplementary material for: Waiting for family reunification and the risk of mental disorders among refugee fathers: a 24-year longitudinal cohort study from Denmark
Source: Soc Psychiatry Psychiatr Epidemiol. 2021 Sep 5;57(5):1061–72. doi: 10.1007/s00127-021-02170-1 (PMC9042990; doi:10.1007/s00127-021-02170-1)
Supplement: Supplementary file 1 — Supplementary file1 (DOCX 2950 KB) [file 127_2021_2170_MOESM1_ESM.docx]

# Supplementary material

## The Danish Context

The number refugees seeking asylum in Denmark has varied markedly during the last 35 years with peaks around 1993 and 2014–2015 following, respectively, the Yugoslav and the Syrian wars [1]. Moreover, in the period 1992–2017 there has been 145 changes to the Danish Aliens Act [1]. Most of these changes have led to more restrictive immigration and integration policies, affecting for instance, the case-processing time of asylum applications, the number of issued residence permits, and the welfare payment for newly resettled refugees [2, 3]. Additionally, the public opinion towards refugees has also turned more negative [4, 5]. As post-resettlement conditions are important for recovery, these changes may have negatively affected the mental health of the refugee fathers [6, 7]. In 2016 (after the period covered by the present study), the Danish government restricted the right to family reunification for refugees with a temporary residence permit. Consequently, some fathers now must wait three years before they can apply for family reunification, prolonging their total family separation period to around four years. Similar changes were introduced in other European countries [8].


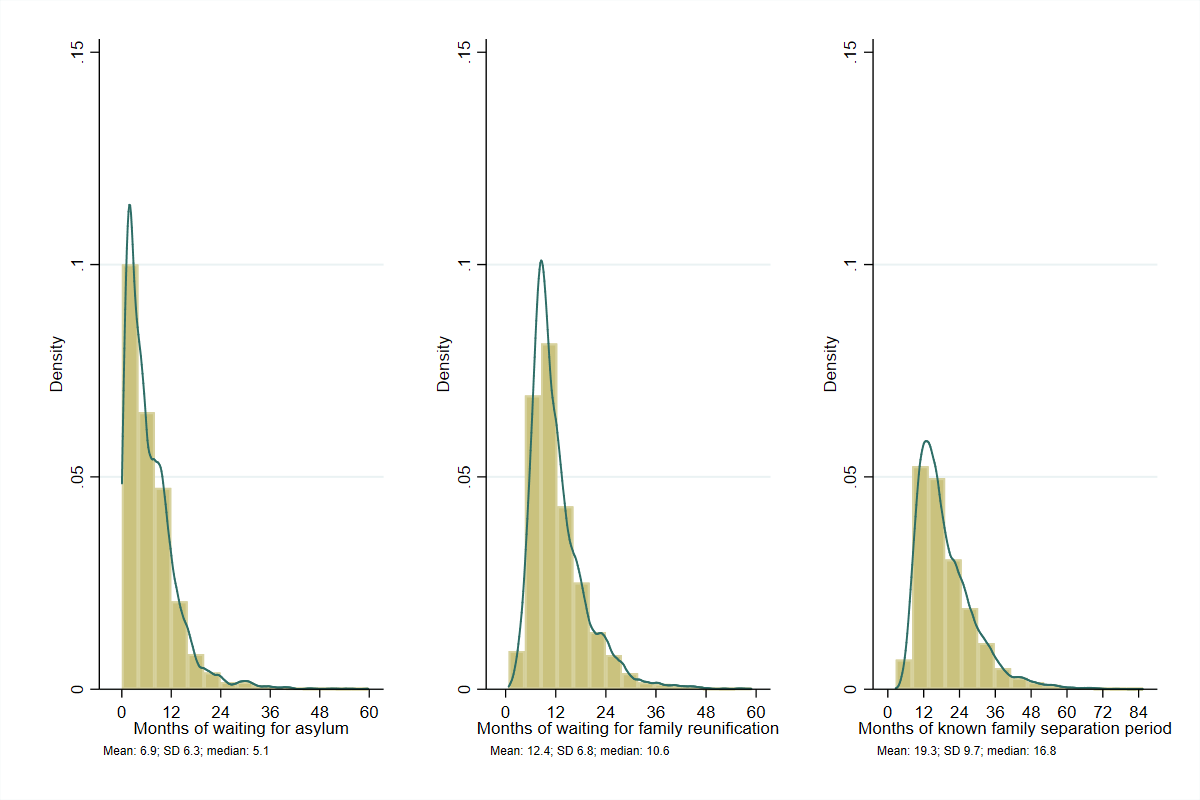


**Supplementary Fig 1** Distributions of the refugee fathers’ time waiting for asylum, time waiting for family reunification, and total family separation period.

**Supplementary Table 1** Distribution (N), person-years (PYs), number of any mental disorder (Diagnoses), and incidence rates (IRs) with 95% confidence intervals across country of origin.

| **Country of origin** | **N** | **PYs** | **Diagnoses** | **IRs** | **95% CI** |
| --- | --- | --- | --- | --- | --- |
| **Syria** | 2785 | 11936 | 388 | 32.5 | 29.4 - 35.9 |
| **Iraq** | 1387 | 20479 | 414 | 20.2 | 18.4 - 22.3 |
| **Afghanistan** | 822 | 11369 | 252 | 22.2 | 19.6 - 25.1 |
| **Iran** | 98 | 815 | 31 | 38.1 | 26.8 - 54.1 |
| **Kuwait** | 71 | 1075 | 18 | 16.7 | 10.5 - 26.6 |
| **Somalia** | 399 | 5216 | 45 | 8.6 | 6.4 - 11.6 |
| **Eritrea** | 324 | 1336 | 5 | 3.7 | 1.6 - 9.0 |
| **Sri Lanka** | 28 | 416 | 8 | 19.2 | 9.6 - 38.4 |
| **Stateless** | 27 | 121 | 9 | 74.5 | 38.8 - 143.1 |
| **Russia** | 18 | 141 | 7 | 49.7 | 23.7 - 104.2 |
| **Bosnia** | 19 | 370 | 7 | 18.9 | 9.0 - 39.7 |
| **Other countries** | 198 | 2669 | 35 | 13.1 | 9.4 - 18.3 |

Diagnoses based on first-time hospital contact for 6176 refugee fathers resettled in Denmark 1995–2015.

**Supplementary Table 2** Distribution (N), person-years (PYs), number of any mental disorder, and incidence rates (IRs), across different lengths of waiting for non-Syrians and Syrians

|  | **Non-Syrians** | | | | **Syrians** | | | |
| --- | --- | --- | --- | --- | --- | --- | --- | --- |
|  | N | PYs | Diagnoses | IRs | N | PYs | Diagnoses | IRs |
| **Family reunification waiting time** |  |  |  |  |  |  |  |  |
| 0–5 months | 418 | 6705 | 93 | 13.9 | 136 | 663 | 16 | 24.1 |
| 6–11 months | 1416 | 19952 | 412 | 20.6 | 1712 | 7549 | 234 | 31.0 |
| 12–60 months | 1557 | 17350 | 326 | 18.8 | 937 | 3723 | 138 | 37.1 |
| **Asylum decision waiting time** |  |  |  |  |  |  |  |  |
| 0–2 months | 185 | 1403 | 23 | 16.4 | 1774 | 7589 | 228 | 30.0 |
| 3–5 months | 603 | 7591 | 125 | 16.5 | 827 | 3393 | 113 | 33.3 |
| 6–11 months | 1703 | 22767 | 423 | 18.6 | 138 | 680 | 31 | 45.6 |
| 12–60 months | 900 | 12246 | 260 | 21.2 | 46 | 274 | 16 | 58.4 |

Diagnoses based on first-time hospital contact for 6176 refugee fathers resettled in Denmark 1995–2015.

**Supplementary Table 3** Hazard ratios of any mental diagnosis, unstratified analysis

| **Variable** | **Categories** | **HRs** | **95% CIs** |
| --- | --- | --- | --- |
| **Family separation** | Reunified (ref.) | 1 | 1 - 1 |
|  | Separated | 2.07*** | 1.56 - 2.75 |
| **Total known family separation period** | 0–8 months (ref.) | 1 | 1 - 1 |
|  | 9–11 months | 1.36* | 1.05 - 1.77 |
|  | 12–17 months | 1.42** | 1.11 - 1.83 |
|  | 18–23 months | 1.78*** | 1.35 - 2.34 |
|  | 24–90 months | 1.61** | 1.21 - 2.13 |
| **Age at application** | 18–29 years (ref.) | 1 | 1 - 1 |
|  | 30–39 years | 1.24* | 1.05 - 1.46 |
|  | 40–65 years | 1.25* | 1.05 - 1.48 |
| **Country of origin** | Syria (ref.) | 1 | 1 - 1 |
|  | Iraq | 1.16 | 0.83 - 1.64 |
|  | Afghanistan | 1.16 | 0.83 - 1.61 |
|  | Iran | 1.29 | 0.84 - 1.96 |
|  | Kuwait | 0.98 | 0.56 - 1.74 |
|  | Somalia | 0.47*** | 0.31 - 0.72 |
|  | Eritrea | 0.08*** | 0.03 - 0.20 |
|  | Sri Lanka | 1.15 | 0.53 - 2.47 |
|  | Stateless | 2.40** | 1.23 - 4.68 |
|  | Russia | 1.61 | 0.73 - 3.58 |
|  | Bosnia | 1.76 | 0.71 - 4.35 |
|  | Other countries | 0.72 | 0.46 - 1.12 |
| **Period of application** | 1991–1995 (ref.) | 1 | 1 - 1 |
|  | 1996–2001 | 1.48 | 0.96 - 2.29 |
|  | 2002–2007 | 2.03* | 1.10 - 3.73 |
|  | 2008–2013 | 3.66*** | 2.25 - 5.96 |
|  | 2014–2015 | 3.03*** | 1.78 - 5.16 |
| **Danish province of resettlement** | Copenhagen, capital (ref.) | 1 | 1 - 1 |
|  | Copenhagen suburb | 1.33 | 0.79 - 2.23 |
|  | North Zealand & Bornholm | 1.69* | 1.12 - 2.55 |
|  | East Zealand | 1.70* | 1.09 - 2.63 |
|  | West & South Zealand | 1.91** | 1.29 - 2.84 |
|  | Funen | 1.48 | 0.98 - 2.24 |
|  | South Jutland | 1.47 | 0.98 - 2.20 |
|  | East Jutland | 1.80** | 1.21 - 2.67 |
|  | West Jutland | 1.63* | 1.08 - 2.44 |
|  | North Jutland | 1.28 | 0.85 - 1.92 |
| **Statistics** | N(observations) | 32835 | |
|  | N(individuals) | 6176 | |
|  | N(diagnoses) | 1219 | |
|  | PH-test (rank) | 85.7 | |
|  | DF (PH-test) | 31 | |
|  | P-value (PH-test) | 0.000 | |

Results are displayed as hazard ratios (HRs) with 95% confidence intervals (CIs). Survival time starts when the fathers receive their residence permit.

* P < 0.05, ** P < 0.01, *** P < 0.001.

**Supplementary Table 7** Hazard ratio for any mental disorder, including variables for education upon arrival

| **Variable** | **Categories** | **HRs** | **95% CIs** |
| --- | --- | --- | --- |
| **Family separation** | Reunified (ref.) | 1.00 | 1.00 - 1.00 |
|  | Separated | 2.06*** | 1.54 - 2.76 |
| **Total known family separation period** | 0–8 months (ref.) | 1.00 | 1.00 - 1.00 |
|  | 9–11 months | 1.39* | 1.05 - 1.84 |
|  | 12–17 months | 1.53** | 1.17 - 2.01 |
|  | 18–23 months | 1.96*** | 1.47 - 2.60 |
|  | 24–90 months | 1.68*** | 1.26 - 2.25 |
| **Education** | No secondary education | 1.00 | 1.00 - 1.00 |
|  | Some education | 0.86* | 0.76 - 0.98 |
| **Imputed/missing education** | Not imputed | 1.00 | 1.00 - 1.00 |
|  | Imputed | 1.51*** | 1.33 - 1.71 |

Results are displayed as hazard ratios (HRs) with 95% confidence intervals (CIs). Survival time starts when the fathers receive their residence permit. Analyses are adjusted for Danish provinces and origin in Middle East or 'other regions', and stratified on age at application (18–29, 30–39, and 40–65 years), period of application (before or after 2001), Sub-Saharan origin, and settlement in province North Jutland.

* P < 0.05, ** P < 0.01, *** P < 0.001.

## References, Supplementary Materials

1. Hvidtfeldt C, Schultz-Nielsen ML (2018) Refugees and asylum seekers in Denmark 1992-2016. Numbers, waiting times, settlement and legislation. The Rockwool Foundation Research Unit

2. Andersen LH, Dustmann C, Landersø R (2019) Lowering Welfare Benefits: Intended and Unintended Consequences for Migrants and their Families. ROCKWOOL Found Res Unit Study Pap No. 138:

3. Arendt JN The effect of welfare benefit reductions on the integration of refugees. 59

4. Czaika M, Lillo AD (2018) The geography of anti-immigrant attitudes across Europe, 2002–2014. J Ethn Migr Stud 44:2453–2479. https://doi.org/10.1080/1369183X.2018.1427564

5. Harmon NA (2018) Immigration, Ethnic Diversity, and Political Outcomes: Evidence from Denmark. Scand J Econ 120:1043–1074. https://doi.org/10.1111/sjoe.12239

6. Hasager L, Jørgensen M (2021) Sick of Your Poor Neighborhood? SSRN Electron J. https://doi.org/10.2139/ssrn.3782634

7. Porter M, Haslam N (2005) Predisplacement and postdisplacement factors associated with mental health of refugees and internally displaced persons: A meta-analysis. JAMA 294:602–612. https://doi.org/10.1001/jama.294.5.602

8. Brekke J-P, Grønningsæter AG (2017) Family reunification regulations in Norway and the EU. Institute for social research
